# Associations between perceived natural environmental features and physical activity intention: the mediating roles of perceived safety and perceived environmental restorativeness

**DOI:** 10.3389/fpubh.2026.1892515

**Published:** 2026-09-10

**Authors:** Wenzhi Yan, Haiying Wang, Mingnan Zhuang, Hanglin Yu, Jinyan Wu, Yunfei Tao, Hansen Li

**Affiliations:** 1 School of Physical Education, Central China Normal University, Wuhan, China; 2 Department of Sports and Exercise Science, Hebei Sport University, Shijiazhuang, China; 3 College of Sports Training, Tianjin University of Sport, Tianjin, China; 4 College of Physical Education, Southwest University, Chongqing, China; 5 School of Sports Training, Chengdu Sport University, Chengdu, Sichuan, China; 6 School of Physical Education, Sichuan Agricultural University, Ya'an, China

**Keywords:** mediation analysis, natural environment, perceived environmental restorativeness, perceived safety, physical activity intention, prospect-refuge theory

## Abstract

**Background:**

Natural environments may support physical activity not only by providing opportunities for movement but also by shaping psychological appraisals of safety and restoration. Guided by prospect-refuge theory, this study distinguished prospect, threat-related refuge, and protective refuge.

**Objective:**

We examined whether perceived safety and perceived environmental restorativeness statistically mediated the associations between these perceived features and physical activity intention.

**Methods:**

A image-based randomized online survey experiment was conducted among 1,439 young adults. Participants were randomly assigned to evaluate one synthetic but realistic natural-landscape image and then completed measures of the three perceived features, perceived safety, the 11-item Perceived Restorativeness Scale (PRS-11), and physical activity intention. Spearman correlations and structural equation modeling with 10,000 bias-corrected bootstrap samples were used, adjusting for age, biological sex, household registration, educational level, and monthly family income.

**Results:**

Prospect and protective refuge were positively associated with physical activity intention, whereas threat-related refuge was negatively associated. The final model showed acceptable fit under commonly used lenient criteria (SRMR = 0.018, TLI = 0.900, CFI = 0.995, RMSEA = 0.064, 90% CI = 0.040 to 0.091). All nine specific indirect associations were statistically significant. For each environmental feature, the pathway through perceived safety alone was the largest specific indirect component in absolute magnitude (prospect: β = 0.151; threat-related refuge: β = −0.096; protective refuge: β = 0.129); the PRS-11-only and serial pathways were smaller. The direct paths to physical activity intention were not statistically significant after the mediators were included, a pattern consistent with indirect-only statistical mediation.

**Conclusions:**

Perceived safety and perceived environmental restorativeness may help explain why prospect, low threat-related concealment, and protective refuge are associated with stronger physical activity intention. Because the mediators and outcome were measured at the same time point, these findings do not establish temporal or causal mediation.

## 1 Introduction

Exposure to natural environments is widely regarded as an important pathway for promoting physical and mental health. Nature contact may influence health through stress reduction, attention restoration, physical activity, social interaction, and improved environmental quality (1–3). For physical activity specifically, environmental features matter not only because they provide physical opportunities but also because aesthetics, facilities, accessibility, and perceived safety influence whether people are willing to enter and use a setting (4). Perceived safety may be a prerequisite for neighborhood green qualities to be associated with physical activity (5). A second research thread indicates that restorative qualities are behaviorally relevant: the restorative quality of exercise settings predicts exercise frequency beyond sociodemographic characteristics, expected benefits, and personal barriers (6), and recent photo-based work links perceived environmental restorativeness with physical activity intention (7). A third thread concerns preference. Environmental preference can represent an expectation that a setting will support effective functioning; when attentional fatigue is greater, people show stronger preference for a restorative walk in nature (8). These findings provide a functional bridge from environmental features to safety, restorative appraisal, preference, and activity. However, the specific spatial cues that initiate this chain have rarely been integrated into one model. We therefore used prospect-refuge theory to examine whether perceived natural spatial features are associated with physical activity intention through perceived safety and perceived environmental restorativeness.

Prospect-refuge theory, originally proposed by Appleton, was developed to explain landscape preference (9). Prospect refers to open views, visual permeability, long-distance visibility, route legibility, and the ability to monitor the surroundings. Refuge refers broadly to shelter, retreat, enclosure, and protection. The theory does not imply that openness is always beneficial or enclosure always harmful; rather, preferred environments often permit people to see without being seen and to move while retaining access to protection. Meta-analytic evidence indicates that prospect and refuge contribute to preference and perceived safety, although their meanings vary by context and appraisal (10). In activity settings, prospect should support surveillance, route prediction, and control, whereas refuge should be beneficial only when it is appraised as usable protection rather than concealment for a threat. This interpretation is supported by previous research. When refuge is perceived as providing shelter or self-protection, it is associated with positive psychological and behavioral responses, such as greater perceived safety, comfort, and environmental preference (11, 12). In contrast, when refuge is perceived as a place of concealment for a potential threat (e.g., a hiding place for an offender), it may evoke negative emotions such as fear and lead to unfavorable evaluations of safety (10, 13). In other words, refuge can be either protective or threatening, depending on how it is operationalized and the context in which it is assessed.

This distinction also clarifies the proposed serial order of the mediators. In broad restoration theory, an environment that continues to signal danger is unlikely to permit attentional disengagement and recovery because vigilance competes with effortless attention. Empirical work has accordingly treated perceived safety or danger as an upstream condition of judged restorative potential (14, 15). After a setting is judged sufficiently safe, its fascination, being-away, compatibility, and scope can be experienced as restorative. Restoration is then behaviorally relevant because people exposed to prolonged demands and directed-attention fatigue seek opportunities to recover; anticipated restoration can make entering, staying in, or being active in a setting worthwhile (6, 8). This ordering therefore reflects a proposed appraisal sequence: spatial cues inform safety, safety permits restorative appraisal, and anticipated restoration supports physical activity intention.

**Figure 1:**
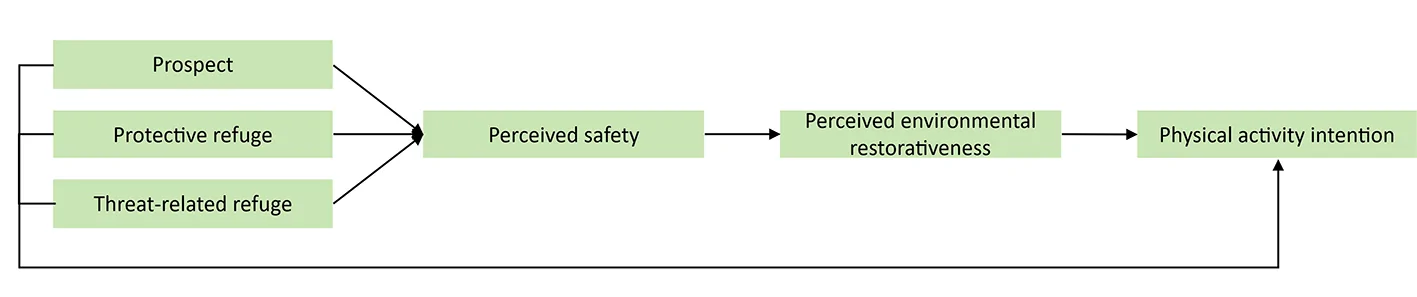
Theoretical model. Single-headed arrows indicate the hypothesized direction of association.

Accordingly, we specified directional hypotheses. H1a: prospect would be positively associated with physical activity intention. H1b: threat-related refuge would be negatively associated with physical activity intention. H1c: protective refuge would be positively associated with physical activity intention. H2a-H2c: each association would also follow a serial indirect pathway through perceived safety and then perceived environmental restorativeness, with positive serial associations for prospect and protective refuge and a negative serial association for threat-related refuge. To distinguish these prespecified long indirect paths from shorter or unanticipated associations, the initial model emphasized the serial structure; direct and shorter paths were subsequently evaluated with explicit theoretical scrutiny (Figure 1).

## 2 Materials and methods

### 2.1 Study design

Following previous environmental psychology studies and considering feasibility, this study adopted a photo-based randomized online survey experiment (7, 16, 17). Each participant evaluated one natural-landscape image randomly selected by the survey platform from the stimulus pool. Randomization applied to image assignment only; the perceived environmental features, perceived safety, perceived environmental restorativeness, and physical activity intention were all self-reported once after exposure. The design therefore combined randomized stimulus assignment with a cross-sectional, observational assessment of the proposed mediation pathways.

### 2.2 Participants

Convenience sampling was used. A recruitment team of 20 members distributed the survey through social networks and available online recruitment channels. Recruitment was open to general adult residents in China, including but not limited to university students. Inclusion criteria were (1) age 18 years or older and (2) no color blindness or severe visual impairment that would prevent recognition of image content. Before beginning, all participants read the online study information and provided electronic informed consent. The Ethics Committee of the School of Physical Education, Southwest University, served as the single reviewing body for the multi-institutional research team and approved recruitment of both students and general adult residents on April 28, 2025 (Approval No. 2023-07-17-007).

### 2.3 Quality control and exclusion criteria

A total of 1,644 questionnaires were collected. Quality control proceeded sequentially. First, 10 questionnaires (0.6% of the initial sample) with invalid or abnormal age entries were excluded, leaving 1,634. Second, completion times were inspected. Consistent with the prespecified empirical rule of reviewing the fastest approximately 5% of responses (18), 86 questionnaires (5.2% of the initial sample) completed in 68 sec or less were excluded; this threshold corresponded to approximately 2 sec per questionnaire item and was judged implausibly fast on inspection. Third, 109 questionnaires (6.6%) were excluded because respondents selected exactly the same response value across every item measured on an 11-point rating scale, including all 11 PRS items and the single-item ratings of prospect, threat-related refuge, protective refuge, perceived safety, and physical activity intention. This pattern was treated as evidence of insufficient-effort responding. In total, 205 questionnaires (12.5%) were excluded, leaving 1,439 participants for analysis. The survey platform displayed a reminder whenever a required response was missing and permitted submission only after all required items had been completed. Consequently, the submitted questionnaires contained no item-level or covariate missing data. These exclusions were applied during data cleaning before the substantive model was estimated; no sensitivity analysis retaining these records was conducted because the invariant response pattern was judged not to provide credible construct-level information.

### 2.4 Image material development

Image development followed the natural-landscape framework proposed by Li et al. (19), which classifies scenes by the presence or absence of three basic element families: plants, water, and rocks/minerals. We included the single-element categories R (rocks/minerals), P (plants), and W (water); the two-element categories WR (water and rocks/minerals), WP (water and plants), and RP (rocks/minerals and plants); and the three-element category WRP (water, rocks/minerals, and plants), yielding seven categories. This coverage was selected to include green, blue, and resource-sparse scenes and to deliberately generate broad variation in prospect, refuge, perceived safety, and perceived environmental restorativeness. The categories therefore served as stimulus-design strata rather than as the independent variables of the mediation model; the analysis focused on participants' continuous appraisals across the pooled scenes.

The original plan was to use photographs of real settings. However, available photographs presented copyright restrictions and substantial incomparability in viewpoint, weather, visual clutter, image quality, people or animals, and other biological content. Collecting a new matched photographic set across widely separated and sometimes extreme environments exceeded the project budget. Realistic images were therefore synthesized with GPT-5.0 as a resource-constrained compromise that allowed cleaner and more standardized scenes. Emerging affective and environmental research has similarly used generative-AI images when tailored or standardized stimuli are difficult to obtain (20, 21). Three design principles were applied: normal eye-level perspective, sunny weather, and visible space in which a person could plausibly stay or be active. Forty candidate images were generated. The research team selected 21 based on apparent realism, consistency with real Chinese natural landscapes, absence of obvious artifacts, and compliance with the design principles. No independent-rater validation was conducted. Supplementary Figure S1 contains the complete 21-image stimulus set, organized by the seven landscape categories, together with the category assignment for each image.

### 2.5 Variables and measures

#### 2.5.1 Perceived environmental features

The following single-item measures were used to operationalize the three perceived features. (1) Prospect: “The place shown in the image has an open view, and everything can be seen clearly.” (2) Threat-related refuge: “Some places in the image look as if they could easily conceal dangerous factors, such as dangerous animals.” (3) Protective refuge: “If danger occurs, such as dangerous animals or natural disasters, I could easily find a place in the image to hide and protect myself.” Each item was rated on an 11-point Likert scale. The protective-refuge item thus operationalized access to refuge specifically as an emergency self-protection affordance; it did not cover all everyday forms of refuge, such as a place to rest or shelter from weather.

#### 2.5.2 Perceived safety

Referring to previous image-based studies (22, 23), perceived safety was measured with a single item: “If you were in the place shown in the image, would you feel safe?” Responses were recorded on an 11-point Likert scale.

#### 2.5.3 Perceived environmental restorativeness

Perceived environmental restorativeness was measured with the 11-item Perceived Restorativeness Scale (PRS-11) originally developed by Pasini et al. (24). The PRS-11 captures being away (3 items), fascination (3 items), compatibility (3 items), and scope (2 items). We administered the revised Chinese version developed and psychometrically evaluated by Li et al. (25). The published Chinese wording was used without further translation, back-translation, or item adaptation by the present research team. Each item was scored from 1 to 11, and the 11-item scores were summed to produce a total score ranging from 11 to 121, with higher scores indicating greater perceived environmental restorativeness. The PRS-11 total score showed acceptable internal consistency in the present sample (Cronbach's alpha = 0.912; McDonald's omega = 0.919).

#### 2.5.4 Physical activity intention

Physical activity intention was measured with one item applied identically across all seven landscape categories: “Would you be willing to engage in physical activity in the environment shown in the image, such as walking or boating (if there is sufficient water)?” Here, setting-compatible physical activity meant movement that could reasonably be performed in the depicted environment; walking represented land-based activity, whereas boating illustrated a possible water-based activity in blue-space scenes. An 11-point numerical rating scale was used (0 = not at all willing; 10 = extremely willing). The unified wording reduced category-specific wording effects, but the measure captured only general willingness and did not distinguish activity intensity, duration, or a specific behavioral plan.

No validated multi-item instruments tailored to rapid image-based assessment of prospect, threat-related refuge, protective refuge, perceived safety, and setting-specific physical activity intention were identified. Single, concrete items were therefore used to limit respondent burden and were modeled as observed variables. Methodological work shows that single indicators can be incorporated into structural path models and may be defensible for narrowly defined, concrete appraisals (26–28). Nevertheless, this choice does not estimate item-specific reliability or separate measurement error from structural relations; accordingly, these paths are interpreted as exploratory associations rather than error-free latent-construct effects.

#### 2.5.5 Participant characteristics

Sociodemographic characteristics included age, biological sex (male = 1; female = 2), household registration (hukou; the Chinese administrative classification of a person's registered rural or urban status, which may differ from current residence; rural = 1, urban = 2), educational level (1 = below undergraduate level; 2 = undergraduate or equivalent; 3 = above undergraduate level), and monthly family income (1 = 0–5,000 CNY; 2 = 5,001–10,000 CNY; 3 = 10,001–15,000 CNY; 4 = 15,001–20,000 CNY; 5 = 20,001–25,000 CNY; 6 = >25,000 CNY).

### 2.6 Statistical analysis

#### 2.6.1 Descriptive analysis

Participant characteristics and study variables were first summarized descriptively. Frequencies and percentages were reported for categorical and ordinal variables. Means and standard deviations were calculated for continuous variables.

#### 2.6.2 Correlation analysis

Spearman correlations were used to describe the correlation pattern among the main variables. Correlation coefficients were interpreted using the following ranges: 0–0.10, negligible; 0.11–0.39, weak; 0.40–0.69, moderate; 0.70–0.89, strong; and 0.90–1.00, very strong (29, 30). The analysis was performed in SPSS version 26.

#### 2.6.3 Mediation analysis

**Figure 2:**
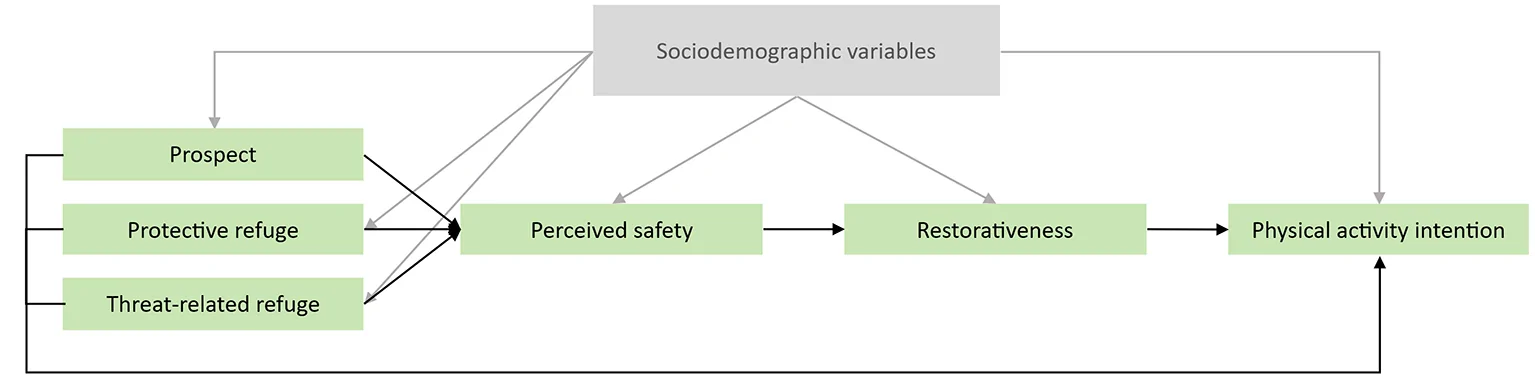
Tested model. Black arrows indicate the focal structural paths, and gray arrows indicate covariate paths from age, biological sex, hukou, educational level, and monthly family income. All arrows point from the predictor to the modeled outcome.

Structural equation modeling (SEM) with conventional maximum-likelihood estimation was used to estimate the proposed direct and indirect associations. Maximum likelihood was selected because the focal observed variables were based on 11-point ratings or the summed PRS-11 score and were treated as approximately continuous, and because the analytic sample was large. Bias-corrected bootstrapping with 10,000 resamples generated bias-corrected 95% confidence intervals for the indirect associations (31, 32). An indirect association whose confidence interval excluded zero was treated as statistically supported. The survey settings prevented incomplete submission, and no item-level or covariate data were missing; therefore, no imputation or full-information maximum likelihood procedure was required, and all 1,439 complete records were analyzed. All sociodemographic characteristics were included as covariates (Figure 2). Neither a robust maximum-likelihood sensitivity analysis nor formal multivariate-normality or model-based influence diagnostics were performed. Accordingly, the conventional maximum-likelihood estimates and global fit indices are interpreted cautiously alongside the complementary fit indices and bootstrap confidence intervals. A formal *a priori* power calculation was not conducted because recruitment was resource-defined and used convenience sampling rather than a probability sample designed to estimate population parameters. As a model-based adequacy check, the common N:q guideline of approximately 10 observations per freely estimated parameter was considered (33). The final model estimated 63 free parameters, requiring approximately 630 observations under this rule; the analytic sample of 1,439 exceeded that benchmark. This rule is only a heuristic, and adequate sample size also depends on effect sizes, distributions, model structure, and estimator performance.

Model fit was evaluated by triangulating indices with different assumptions and penalties rather than treating any single cutoff as decisive. The more stringent reference values were SRMR < 0.08, TLI and CFI > 0.95, and RMSEA < 0.05; TLI > 0.90 and RMSEA < 0.10 were also treated as commonly used lenient indicators of acceptable, rather than good, fit (34, 35). RMSEA is reported with its 90% confidence interval. Variance inflation factors (VIFs) were obtained from an auxiliary multiple linear regression in which physical activity intention was the dependent variable and age, biological sex, educational level, monthly family income, household registration, prospect, threat-related refuge, perceived safety, protective refuge, and the PRS-11 score were entered simultaneously as predictors. Age was entered as a continuous variable; biological sex and hukou were entered as binary numeric variables; and educational level and monthly family income were entered as ordinal numeric variables using the coding specified in Section 2.5.5; no dummy variables were created. Fixed universal VIF cutoffs were interpreted cautiously (36, 37). The VIF values were 1.187 for age, 1.037 for biological sex, 1.203 for educational level, 1.110 for monthly family income, 1.080 for rural-urban household registration, 1.353 for prospect, 1.014 for threat-related refuge, 2.044 for perceived safety, 2.061 for protective refuge, and 1.546 for the PRS-11 score. All VIF values were below 5.0, indicating no serious multicollinearity among the predictors.

#### 2.6.4 Common method variance assessment

Because all substantive variables were collected by self-report in the same questionnaire, Harman's single-factor diagnostic was used as a *post hoc* assessment of common method variance. All substantive item responses were entered into an unrotated exploratory factor analysis. We recorded the number of factors with eigenvalues greater than 1 and the percentage of total variance explained by the first factor. A first-factor share below 50% was treated as evidence against one dominant common-method factor (38), while recognizing that this diagnostic cannot rule out common method bias. The analysis was performed in SPSS version 26.

#### 2.6.5 Sensitivity analyses

Sensitivity analyses assessed whether the structural paths were materially different across biological-sex and household-registration groups. For each grouping variable, a multigroup model with structural paths freely estimated was compared with a model in which the structural paths were constrained equal across groups. A change in CFI (ΔCFI) smaller than 0.010 was interpreted as indicating no practically important deterioration after imposing equality constraints (39, 40). Because the PRS-11 was entered as an observed total score, item-level configural, metric, and scalar measurement invariance was not tested before the structural comparisons. The multigroup results were therefore treated as exploratory sensitivity checks rather than definitive tests of moderation. Education-stratified SEM was not undertaken because the below-undergraduate and above-undergraduate groups were small relative to the undergraduate/equivalent group, which could produce unstable estimates for the present model. The SEM-related analyses were conducted using AMOS version 28.

#### 2.6.6 Exploratory factor analysis of the PRS-11

Because the PRS-11 was administered in Chinese and its total score was used in the structural model, a supplementary exploratory factor analysis (EFA) was conducted to examine its dimensionality in the present sample. COSMIN considers both exploratory and confirmatory factor analysis useful for evaluating structural validity: EFA is rated as an adequate method, whereas confirmatory factor analysis (CFA) is preferred when testing an *a priori* factor structure (41). Accordingly, the present EFA was used as a supplementary, data-driven assessment of the Chinese-language item responses rather than as definitive confirmation of the original PRS-11 structure. The 11-category item responses were treated as approximately continuous. Sampling adequacy was assessed with the Kaiser-Meyer-Olkin (KMO) statistic and Bartlett's test of sphericity. Common-factor analysis was performed using principal axis factoring on the Pearson correlation matrix rather than principal component analysis. The number of factors was determined using parallel analysis with 1,000 random datasets and the 95th-percentile criterion, considered together with the scree pattern and eigenvalues. An oblique rotation was planned if more than one factor was retained; no rotation was applied to a one-factor solution. Factor loadings, communalities, Cronbach's alpha, and McDonald's omega were reported. The analysis was conducted in Python 3.12 with NumPy 2.3.5. A *post hoc* item-deletion sensitivity analysis was then conducted for PRS3, the only item with a factor loading below 0.30. Pearson and Spearman correlations were used to compare the original 11-item total with a 10-item score excluding PRS3. Because the two raw totals have different possible ranges, both scores were also expressed as per-item means and evaluated using Lin's concordance correlation coefficient and Bland-Altman mean differences with 95% limits of agreement. Cronbach's alpha was recalculated after excluding PRS3. The 10-item score was treated as a *post hoc* sensitivity score rather than a replacement for the primary PRS-11 score. Retaining the established 11-item scoring preserves correspondence with the wider PRS-11 literature, particularly studies using the revised Chinese PRS-11 validated by Li et al. (25); the empirical agreement between the 11-item and 10-item scores was evaluated as an additional robustness check.

## 3 Results

### 3.1 Participant characteristics

Of the 1,644 questionnaires collected, 205 were excluded during sequential quality control and 1,439 were retained (Table 1). The analytic sample included 350 men (24.3%) and 1,089 women (75.7%); 1,032 participants (71.7%) had rural household registration and 407 (28.3%) had urban household registration. Most participants were undergraduate students or had an equivalent educational level (*n* = 1,204, 83.7%). Monthly family income was concentrated in the 0–5,000 CNY (*n* = 549, 38.2%) and 5,001–10,000 CNY (*n* = 567, 39.4%) categories. The mean PRS-11 score was 76.00 (SD = 18.28), and the mean scores for prospect, threat-related refuge, protective refuge, perceived safety, and physical activity intention were 7.45 (SD = 2.26), 7.03 (SD = 2.39), 4.93 (SD = 2.39), 6.07 (SD = 2.47), and 7.03 (SD = 2.64), respectively.

**Table 1:** Sociodemographic profile and descriptive statistics of the analytic sample (*N* = 1,439).

Variable	Category	*n* (%)	Mean (SD)
Biological sex	Male	350 (24.3)	–
	Female	1,089 (75.7)	–
Household registration	Rural	1,032 (71.7)	–
	Urban	407 (28.3)	–
Educational level	Below undergraduate level	122 (8.5)	–
	Undergraduate or equivalent	1,204 (83.7)	–
	Above undergraduate level	113 (7.9)	–
Monthly family income	0–5,000 CNY	549 (38.2)	–
	5,001–10,000 CNY	567 (39.4)	–
	10,001–15,000 CNY	193 (13.4)	–
	15,001–20,000 CNY	73 (5.1)	–
	20,001–25,000 CNY	26 (1.8)	–
	>25,000 CNY	31 (2.2)	–
Age	–	–	20.51 (2.32)
PRS-11 score	–	–	76.00 (18.28)
Prospect	–	–	7.45 (2.26)
Threat-related refuge	–	–	7.03 (2.39)
Protective refuge	–	–	4.93 (2.39)
Perceived safety	–	–	6.07 (2.47)
Physical activity intention	–	–	7.03 (2.64)

### 3.2 Correlation analysis

The Spearman correlation results are shown in Table 2. Prospect was moderately and positively correlated with perceived environmental restorativeness (ρ = 0.506, *p* < 0.001), perceived safety (ρ = 0.426, *p* < 0.001), and physical activity intention (ρ = 0.435, *p* < 0.001). Threat-related refuge was weakly and negatively correlated with perceived safety (ρ = −0.230, *p* < 0.001) and physical activity intention (ρ = −0.134, *p* < 0.001), but its correlation with perceived environmental restorativeness was not statistically significant (ρ = −0.049, *p* = 0.064). Protective refuge was weakly to moderately and positively correlated with perceived environmental restorativeness (ρ = 0.300, *p* < 0.001), perceived safety (ρ = 0.343, *p* < 0.001), and physical activity intention (ρ = 0.235, *p* < 0.001). Perceived environmental restorativeness and perceived safety were both moderately and positively correlated with physical activity intention (ρ = 0.681 and ρ = 0.673, respectively; both *p* < 0.001). The three perceived environmental features were not significantly correlated with one another, suggesting that prospect, threat-related refuge, and protective refuge captured relatively distinct perceptual dimensions in the present image set.

**Table 2:** Spearman correlations among perceived environmental features, proposed mediators, and physical activity intention (*N* = 1,439).

Variable	Prospect	Threat-related refuge	Protective refuge	PRS-11	Perceived safety	PA intention
Prospect	1.000	–	–	–	–	–
Threat-related refuge	−0.032	1.000	–	–	–	–
Protective refuge	0.048	−0.007	1.000	–	–	–
PRS-11	0.506^**^	−0.049	0.300^**^	1.000	–	–
Perceived safety	0.426^**^	−0.230^**^	0.343^**^	0.643^**^	1.000	–
PA intention	0.435^**^	−0.134^**^	0.235^**^	0.681^**^	0.673^**^	1.000

*N* = 1,439. ^**^p < 0.001. PRS-11 = 11-item Perceived Restorativeness Scale; PA intention, physical activity intention.

### 3.3 Mediation analysis

**Figure 3:**
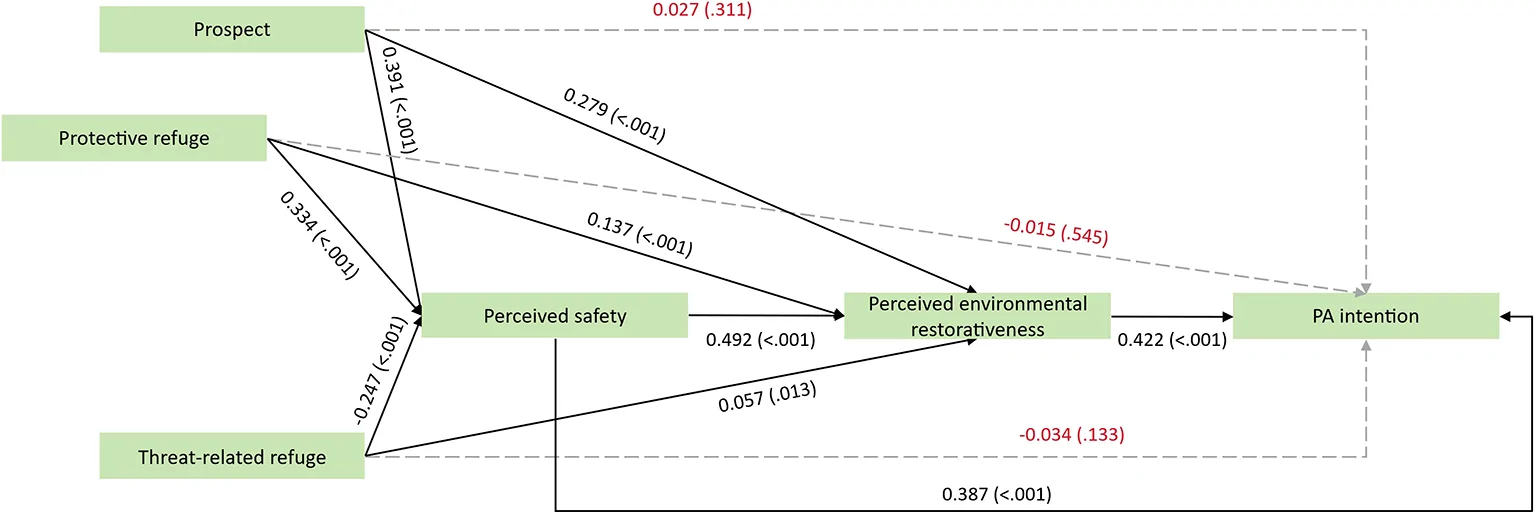
Final model structure. Single-headed arrows indicate the modeled direction of association. Black solid paths are statistically significant; gray dashed paths are non-significant. All paths were adjusted for age, biological sex, hukou, educational level, and monthly family income. Coefficients outside parentheses are standardized regression coefficients; values in parentheses are *p* values.

The original, deliberately constrained model represented the prespecified long serial pathways and showed poor fit (SRMR = 0.051, TLI = −0.097, CFI = 0.860, RMSEA = 0.211; AIC = 574.218; BIC = 885.255). Because the original model omitted several theoretically plausible shorter paths, the modification indices (MIs) from this model were inspected *post hoc* together with the unstandardized expected parameter changes (Par Change). Four paths were added sequentially in descending order of their initial MI values: prospect to PRS-11 score (MI = 116.801, Par Change = 1.749), perceived safety to physical activity intention (MI = 106.034, Par Change = 0.211), protective refuge to PRS-11 score (MI = 28.146, Par Change = 0.812), and threat-related refuge to PRS-11 score (MI = 16.369, Par Change = 0.622). No paths were removed (Figure 3). Although the paths were entered in MI order, their inclusion was also consistent with the theoretical possibility that environmental features may contribute directly to restorative appraisal and that perceived safety may influence activity intention independently of restorativeness. Because all four paths were identified and evaluated in the same sample, they were treated as *post hoc* exploratory paths requiring independent confirmation. The revised model fit improved (SRMR = 0.018, TLI = 0.900, CFI = 0.995, RMSEA = 0.064, 90% CI = 0.040 to 0.091; AIC = 146.615; BIC = 478.732). Both AIC and BIC were markedly lower for the revised model, indicating better relative fit than the original specification. The strong CFI and SRMR contrasted with a borderline TLI and an RMSEA above the stringent 0.05 benchmark. This discrepancy is possible because the indices use different baselines and penalties, with TLI more strongly penalizing model complexity. We therefore describe the model as showing acceptable fit under commonly used lenient criteria, not as good fit, and interpret the structural estimates cautiously.

All nine specific indirect paths from the perceived environmental features to physical activity intention were statistically significant (Table 3). For prospect, the pathway through perceived safety alone was the largest specific indirect component (β = 0.151, 95% CI = 0.124 to 0.183, *p* < 0.001), followed by the PRS-11-only pathway (β = 0.118, 95% CI = 0.092 to 0.148, *p* < 0.001) and the serial pathway through perceived safety and PRS-11 (β = 0.081, 95% CI = 0.067 to 0.098, *p* < 0.001). For protective refuge, the safety-only pathway was likewise the largest (β = 0.129, 95% CI = 0.104 to 0.160, *p* < 0.001), followed by the serial pathway (β = 0.069, 95% CI = 0.056 to 0.086, *p* < 0.001) and the PRS-11-only pathway (β = 0.058, 95% CI = 0.037 to 0.081, *p* < 0.001). For threat-related refuge, the negative safety-only pathway had the largest absolute magnitude (β = −0.096, 95% CI = −0.122 to −0.073, *p* < 0.001), followed by the negative serial pathway (β = −0.051, 95% CI = −0.067 to −0.039, *p* < 0.001); the PRS-11-only pathway was smaller and positive (β = 0.024, 95% CI = 0.005 to 0.044, *p* = 0.013). Thus, perceived safety alone represented the largest specific indirect component for all three environmental features, while the opposing components for threat-related refuge indicated an inconsistent indirect pattern.

**Table 3:** Specific indirect effects from perceived environmental features to physical activity intention.

Path	Estimate	Lower 95% CI	Upper 95% CI	*p*
Prospect → PRS-11 → PA intention	0.118	0.092	0.148	< 0.001
Threat-related refuge → PRS-11 → PA intention	0.024	0.005	0.044	0.013
Protective refuge → PRS-11 → PA intention	0.058	0.037	0.081	< 0.001
Prospect → Perceived safety → PA intention	0.151	0.124	0.183	< 0.001
Threat-related refuge → Perceived safety → PA intention	−0.096	−0.122	−0.073	< 0.001
Protective refuge → Perceived safety → PA intention	0.129	0.104	0.160	< 0.001
Prospect → Perceived safety → PRS-11 → PA intention	0.081	0.067	0.098	< 0.001
Threat-related refuge → Perceived safety → PRS-11 → PA intention	−0.051	−0.067	−0.039	< 0.001
Protective refuge → Perceived safety → PRS-11 → PA intention	0.069	0.056	0.086	< 0.001

All coefficients are standardized. All paths were adjusted for age, biological sex, household registration, educational level, and monthly family income. PRS-11, 11-item Perceived Restorativeness Scale; PA intention, physical activity intention. Bias-corrected bootstrap 95% confidence intervals based on 10,000 resamples are the primary uncertainty estimates; separate normal-theory standard errors are not reported because bias-corrected intervals may be asymmetric.

The total effects of all three perceived environmental features on perceived environmental restorativeness were statistically significant (Table 4). Prospect showed a positive total effect on perceived environmental restorativeness (β = 0.471, 95% CI = 0.420 to 0.518, *p* < 0.001), with both direct and indirect effects being positive and significant. Protective refuge also showed a positive total effect on perceived environmental restorativeness (β = 0.302, 95% CI = 0.250 to 0.352, *p* < 0.001), again with significant direct and indirect components. By contrast, threat-related refuge showed a negative total effect on perceived environmental restorativeness (β = −0.065, 95% CI = −0.118 to −0.015, *p* = 0.012). Its indirect effect through perceived safety was negative (β = −0.122, 95% CI = −0.152 to −0.095, *p* < 0.001), whereas its direct effect on perceived environmental restorativeness was positive (β = 0.057, 95% CI = 0.012 to 0.100, *p* = 0.013). This opposite direction between the direct and indirect effects suggests an inconsistent mediation or suppression pattern for the association between threat-related refuge and perceived environmental restorativeness.

**Table 4:** Direct, indirect, and total effects from perceived environmental features to perceived environmental restorativeness.

Effect	Estimate	Lower 95% CI	Upper 95% CI	*p*
Prospect → PRS-11, total effect	0.471	0.420	0.518	< 0.001
Prospect → PRS-11, indirect effect	0.192	0.164	0.223	< 0.001
Prospect → PRS-11, direct effect	0.279	0.225	0.331	< 0.001
Threat-related refuge → PRS-11, total effect	−0.065	−0.118	−0.015	0.012
Threat–related refuge → PRS-11, indirect effect	−0.122	−0.152	−0.095	< 0.001
Threat-related refuge → PRS-11, direct effect	0.057	0.012	0.100	0.013
Protective refuge → PRS-11, total effect	0.302	0.250	0.352	< 0.001
Protective refuge → PRS-11, indirect effect	0.164	0.136	0.196	< 0.001
Protective refuge → PRS-11, direct effect	0.137	0.089	0.185	< 0.001

All coefficients are standardized. All paths were adjusted for age, biological sex, household registration, educational level, and monthly family income. PRS-11, 11-item Perceived Restorativeness Scale.

Finally, the global indirect associations linking the three environmental features to physical activity intention were examined (Table 5). Prospect had a positive total association with physical activity intention (β = 0.378, 95% CI = 0.319 to 0.432, *p* < 0.001) and a positive indirect association (β = 0.350, 95% CI = 0.313 to 0.390, *p* < 0.001); its direct path was not significant after the mediators were included (β = 0.027, *p* = 0.311). Threat-related refuge had a negative total association (β = −0.157, 95% CI = −0.210 to −0.103, *p* < 0.001) and a significant negative indirect association (β = −0.123, 95% CI = −0.163 to −0.086, *p* < 0.001), whereas its direct path was non-significant (β = −0.034, *p* = 0.133). Protective refuge had a positive total association (β = 0.241, 95% CI = 0.187 to 0.294, *p* < 0.001) and a positive indirect association (β = 0.257, 95% CI = 0.218 to 0.298, *p* < 0.001), with a small non-significant direct path in the opposite direction (β = −0.015, *p* = 0.545). These direct, specific indirect, global indirect, and total associations are reported separately rather than converted into proportions mediated, because ratios are difficult to interpret when direct and indirect components have different signs.

**Table 5:** Direct, indirect, and total effects from perceived environmental features to physical activity intention.

Effect	Estimate	Lower 95% CI	Upper 95% CI	*p*
Prospect → PA intention, total effect	0.378	0.319	0.432	< 0.001
Prospect → PA intention, indirect effect	0.350	0.313	0.390	< 0.001
Prospect → PA intention, direct effect	0.027	−0.025	0.079	0.311
Threat-related refuge → PA intention, total effect	−0.157	−0.210	−0.103	< 0.001
Threat-related refuge → PA intention, indirect effect	−0.123	−0.163	−0.086	< 0.001
Threat-related refuge → PA intention, direct effect	−0.034	−0.076	0.010	0.133
Protective refuge → PA intention, total effect	0.241	0.187	0.294	< 0.001
Protective refuge → PA intention, indirect effect	0.257	0.218	0.298	< 0.001
Protective refuge → PA intention, direct effect	−0.015	−0.062	0.032	0.545

All coefficients are standardized. All paths were adjusted for age, biological sex, household registration, educational level, and monthly family income. PA intention, physical activity intention.

### 3.4 Common method variance assessment

The unrotated factor analysis identified six factors with eigenvalues greater than 1. The first factor explained 33.86% of the total variance, which was below the 50% diagnostic threshold.

### 3.5 Sensitivity analyses

For biological sex, the unconstrained multigroup model showed acceptable fit indices (TLI = 0.923, CFI = 0.995, RMSEA = 0.044, 90% CI = 0.025 to 0.064), and CFI was 0.995 in the equality-constrained model (ΔCFI < 0.001). For household registration, the unconstrained multigroup model showed acceptable fit indices (TLI = 0.901, CFI = 0.993, RMSEA = 0.049, 90% CI = 0.031 to 0.069), and CFI was 0.990 in the equality-constrained model (ΔCFI = 0.003). Under the constraints and sample structure examined, no statistically or practically substantial subgroup differences were detected. Because item-level measurement invariance of the PRS-11 was not established, these findings are interpreted as exploratory sensitivity results rather than evidence that moderation is absent.

### 3.6 Exploratory factor analysis of the PRS-11

The PRS-11 item matrix was suitable for factor analysis [KMO = 0.928; Bartlett's χ^2^(55) = 10,426.815, *p* < 0.001]. Parallel analysis supported one factor: the first observed eigenvalue was 6.182, exceeding the corresponding 95th-percentile random eigenvalue of 1.177, whereas the second observed eigenvalue was 1.052, below the corresponding random value of 1.130. The one-factor principal-axis solution explained 52.6% of the total item variance. Ten items had loadings from 0.513 to 0.869, whereas PRS3 had a lower loading of 0.203 and communality of 0.041 (Supplementary Table S1). Internal consistency was high (Cronbach's alpha = 0.912; McDonald's omega = 0.919). These findings indicate a predominant general restorativeness factor and provide qualified support for use of the total score in this sample, although the weak loading of PRS3 prevents the EFA from being interpreted as definitive evidence of structural validity. A *post hoc* item-deletion sensitivity analysis showed that the original 11-item total and the 10-item score excluding PRS3 were almost perfectly correlated (Pearson's r = 0.991; Spearman's rho = 0.993; both *p* < 0.001). After both scores were placed on a common per-item metric, agreement remained very high (Lin's concordance correlation coefficient = 0.988). The 11-item per-item mean was 0.090 points lower on average than the 10-item per-item mean, with 95% limits of agreement from −0.574 to 0.394. Cronbach's alpha increased from 0.912 to 0.929 after PRS3 was excluded. Thus, retaining PRS3 had little influence on participant rank ordering or the score expressed on a common per-item scale. On the basis of both this empirical stability and the need to preserve direct comparability with previous PRS-11 studies, particularly studies using the revised Chinese PRS-11 validated by Li et al. (25), the established 11-item total was retained for the primary structural analyses.

## 4 Discussion

### 4.1 Summary of study design and main findings

Guided by prospect-refuge theory, this image-based randomized online survey experiment examined associations between perceived natural environmental features and physical activity intention, together with the statistical mediating roles of perceived safety and perceived environmental restorativeness. Prospect and protective refuge were positively associated with physical activity intention, whereas threat-related refuge was negatively associated. Across the nine specific indirect pathways, the pathway through perceived safety alone was the largest component for prospect and protective refuge and the largest component in absolute magnitude for threat-related refuge. The PRS-11-only and serial pathways were also statistically significant, but smaller. Once perceived safety and the PRS-11 score were included, the direct paths to physical activity intention were not statistically significant. This indirect-only statistical pattern is consistent with the proposed appraisal sequence, but it does not establish that safety and restoration are the only mechanisms or that the ordering is causal.

The findings suggest that people may be more willing to be active where spatial cues support a sense of control and anticipated restoration. Clear views can facilitate monitoring and route prediction, protective refuge can provide a usable fallback, and threat-related concealment can sustain vigilance. Perceived safety is therefore theoretically positioned before restorative appraisal, while anticipated restoration supplies a reason to enter or remain active in the setting. At the same time, the non-significant direct paths should not be treated as proof of full or causal mediation: limited reliability, shared-method variance, omitted variables, or alternative path orderings could also contribute to this pattern.

Threat-related refuge showed a negative safety-mediated association with perceived environmental restorativeness but a small positive direct path after perceived safety was controlled. Its zero-order correlation with the PRS-11 score was also non-significant. These results can coexist when opposing components cancel in the bivariate association: concealment may reduce safety and thereby undermine restoration, whereas residual features correlated with concealment, such as benign enclosure, mystery, or organized visual complexity, may weakly support fascination after safety is held constant. Attention Restoration Theory describes soft fascination as effortless engagement, and empirical work links complexity and mystery with restorative appraisal (42–44). The positive direct coefficient should therefore not be read as evidence that danger is restorative. Instead, it suggests that the threat-related-refuge item may combine a negative threat appraisal with visually engaging spatial structure. Future measures should separate concealment-induced threat from benign enclosure, mystery, and complexity. The negative total association and negative path through safety remain the dominant pattern.

The initial model intentionally prioritized the hypothesized long serial chain and omitted several shorter paths, making it highly constrained. Theory-guided *post hoc* respecification is used in applied SEM when an *a priori* model does not capture plausible relations; published examples have combined modification indices with substantive justification to add or remove paths (45–47). Nevertheless, the four added paths were informed by the covariance pattern in the same sample and may capitalize on sample-specific associations; no split-sample calibration-validation analysis was conducted. The biological-sex and household-registration multigroup analyses yielded ΔCFI < 0.001 and ΔCFI = 0.003, respectively, after equality constraints were imposed on the structural paths, providing only a limited assessment of path stability across these subgroups. Item-level measurement invariance of the PRS-11 was not established before these comparisons. These subgroup analyses are neither out-of-sample validation nor definitive tests of moderation. We therefore distinguish the prespecified serial paths from the added exploratory paths and regard replication or cross-validation in an independent sample as necessary.

The supplementary EFA suggested that a general restorativeness factor dominated the PRS-11 responses in this sample, which provides preliminary, qualified support for use of the total score. However, PRS3 was weakly related to that factor and remains an item-level measurement concern. Our decision to retain the established 11-item total rather than replace it with the *post hoc* 10-item score rested on two complementary considerations. First, excluding PRS3 left participant rank ordering essentially unchanged (Pearson's r = 0.991; Spearman's rho = 0.993), produced very high agreement on a common per-item metric (Lin's concordance correlation coefficient = 0.988), and increased reliability only modestly from 0.912 to 0.929. Second, retaining the complete PRS-11 preserves direct correspondence with the wider literature using this scale, especially studies using the revised Chinese PRS-11 validated by Li et al. (25). This comparability is particularly important because the present study analyzed overall restorativeness rather than dimension-specific scores. The near-perfect correlations should nevertheless be interpreted as part-whole associations because the two scores share 10 items; they do not establish literal interchangeability of raw sums with different ranges. Retaining the 11-item total also does not resolve the weak performance of PRS3. Because the PRS-11 has an *a priori* multidimensional structure, item-wording and translation review and CFA in an independent sample remain necessary.

### 4.2 Comparison with related studies

The finding that prospect may increase physical activity intention by enhancing perceived safety and perceived environmental restorativeness aligns with studies of visibility and perceived danger in forest environments. Herzog and Kirk (48) found that border visibility and visual access in forest path scenes were positively associated with preference and negatively associated with perceived danger, and that danger perception was more strongly influenced by visibility-related factors than preference was. This suggests that, in natural environments, clear views and recognizable spatial boundaries influence not only aesthetic preference but also judgments of potential risk. The present study extends this evidence by showing that prospect may not directly promote physical activity intention; rather, it appears to operate primarily by enhancing perceived safety and restorative experience.

The findings regarding threat-related refuge also correspond with studies of urban forests and public open spaces. Wang et al. (49) found that visual quality, facility completeness, and accessibility positively predicted tourists' safety perception in urban forests in Fuzhou, China, with safety emotion and sense of control playing important mediating roles in safety-environment evaluation. This indicates that safety in natural spaces does not simply depend on the presence of natural elements; it also depends on whether individuals can perceive the environment clearly, judge risks, and form a sense of control. Ríos-Rodríguez et al. (50) similarly found that design quality, maintenance, social interaction, and sensory elements in public spaces explained variation in perceived environmental restorativeness, indicating that restorativeness is shaped by both spatial organization and environmental quality. The present findings add to this view by showing that when refuge is interpreted as potential concealment for danger, it can undermine perceived safety and subsequently reduce restorativeness and physical activity intention.

Importantly, protective refuge was positively associated with physical activity intention, whereas threat-related refuge was negatively associated with physical activity intention. This indicates that refuge in natural environments is not a single-direction psychological cue; rather, its meaning depends on whether it is interpreted as a protective resource or a source of risk. This interpretation is consistent with research on environmental quality and restorativeness: the effect of a natural or semi-natural space depends not only on the amount of nature but also on how natural elements are organized into a legible, usable, and reassuring environment. Moderate shelter, boundaries, and places for rest may provide protection and opportunities for staying, whereas overly dense vegetation, blind spots, or unclear routes may be perceived as potential sources of threat.

The present results also align with recent studies on the pathway from environmental perception to restorativeness and physical activity. Zhang et al. (51) found among Chengdu residents that perceived environmental quality of urban green space was positively associated with physical activity level and that perceived environmental restorativeness partially mediated this association. Li et al. (52) also showed that subjectively perceived nighttime lighting was related to perceived safety, perceived environmental restorativeness, and nighttime physical activity, with perceived environmental restorativeness serving as a key mediator between subjective lighting and nighttime physical activity. Together with the present study, these findings suggest that physical activity is not determined only by objective environmental resources. People's subjective interpretations of environmental quality, safety, and restoration may also be central mechanisms.

Finally, previous research has shown that physical activity in natural environments often provides stronger restorative experiences than activity in indoor or built environments. Pasanen et al. (53) found that everyday physical activity in natural settings was associated with higher restorative experiences. Laezza et al. (54), in a randomized controlled trial, also showed that light-to-moderate exercise in natural environments produced greater relaxation, pleasure, and stress recovery than exercise in urban or indoor environments. In contrast to these studies, the present study focused not on differences between natural and non-natural environments, but on how different perceived spatial features within natural environments shape physical activity intention. The study therefore advances the broader conclusion that nature can support activity and restoration by showing that natural spaces may be more likely to encourage physical activity when they simultaneously provide clear views, appropriate protection, low threat, and high perceived environmental restorativeness.

### 4.3 Contributions

This study makes several contributions. First, at the theoretical level, it introduced prospect-refuge theory into research on natural environments and physical activity intention and distinguished refuge into protective refuge and threat-related refuge. This distinction helps explain why the direction of refuge effects has been inconsistent in prior research. Shelter, enclosure, and boundaries in natural environments do not necessarily reduce perceived safety or restorativeness. When these features are perceived as providing protection, rest, or self-regulation, they may promote restorativeness and physical activity intention; when they are perceived as concealing danger, they may have the opposite effect.

Second, at the mechanistic level, the study constructed and tested a serial pathway from perceived environmental features to perceived safety, perceived environmental restorativeness, and physical activity intention. The results indicated that perceived safety and perceived environmental restorativeness play central mediating roles in the associations between the three environmental features and physical activity intention. This suggests that the influence of environmental design on physical activity intention is not a simple physical stimulus-behavior response process, but involves psychological processing such as safety appraisal and restorative evaluation. This finding helps connect environmental psychology, restorative-environment research, and physical-activity promotion.

Third, at the practical level, the findings offer implications for the design of natural spaces, campus landscapes, park trails, and outdoor activity settings. To increase people's willingness to be physically active in natural environments, design should not focus only on the quantity of greenery, water, or natural elements. It should also consider visibility, route clarity, boundary organization, and the construction of perceived safety. For example, improving visual openness, reducing blind spots, avoiding excessively dense vegetation along key routes, and preserving tree shade, resting points, and semi-enclosed protective spaces may jointly enhance perceived safety, restorativeness, and physical activity intention. In this sense, an ideal natural activity space should balance the ability to “see clearly” with the feeling of “being protected.”

The seven landscape categories were used to generate perceptual diversity rather than to estimate category-specific effects. Accordingly, the practical implications concern perceived prospect, threat-related concealment, protection, safety, and restoration across the pooled scenes; they should not be interpreted as evidence that any one element-based category is uniformly superior. Translating the findings into category-specific planning strategies requires a separate analysis that directly models objective landscape composition and activity affordances.

### 4.4 Limitations

First, the static online image context has limited ecological validity. Participants likely completed the survey in comfortable indoor settings, where judging danger carries fewer immediate consequences than being physically present. Static images omit temperature, humidity, sound, smell, bodily movement, and changing weather or social conditions; these cues may affect prospect, refuge, safety, and perceived environmental restorativeness. The PRS-11 being-away dimension may be especially constrained because participants did not actually leave their everyday setting. Real-world field experiments, immersive multisensory simulations, and walking studies are needed.

Second, AI-generated stimuli were a pragmatic response to copyright, comparability, and budget constraints, but no independent panel validated their realism. Subtle artifacts, impossible spatial configurations, idealized composition, or research-team selection bias could systematically alter psychological responses and limit comparison with photograph-based studies. In addition, natural terrain and ecosystem structure made visible activity space difficult to match perfectly; the usable space in some WRP scenes may have been less recognizable than in other scenes. Because perceived activity space is related to prospect, safety, and willingness to move, it may be an uncontrolled confound. Future work should obtain independent ratings of realism and naturalness, compare AI images with matched photographs and real sites, and quantify visible, walkable, and water-based activity affordances.

Third, the outcome was a single general measure of physical activity intention rather than behavior. Walking and boating were included to cover land and blue-space possibilities, and identical wording reduced wording-related differences across categories. However, the activities available in natural environments vary in intensity, duration, skill, feasibility, and health benefit (55, 56). Participants may therefore have imagined different activities, and water, terrain, or visible space may itself have influenced willingness. The results concern general setting-specific willingness, not a defined activity dose or actual participation. Future studies should measure activity type, duration, intensity, and behavior with GPS, accelerometry, or observation.

Fourth, the convenience sample was young, predominantly female (75.7%), predominantly rural-hukou (71.7%), and educationally homogeneous (83.7% undergraduate or equivalent). Sex, age, education, familiarity, and physical capacity may change threat appraisal, perceived safety, restoration, and activity choice. The sex and hukou multigroup sensitivity analyses provide a limited check, but the small below-undergraduate and above-undergraduate groups prevented a stable education-stratified model. Convenience recruitment also creates selection bias that a large sample does not remove. Generalization to men, older adults, children, non-students, and people with more varied educational backgrounds should therefore be made cautiously.

Fifth, prospect, threat-related refuge, protective refuge, perceived safety, and physical activity intention were measured with single items because suitable validated multi-item tools were unavailable. Single items cannot assess internal consistency and may underrepresent multidimensional constructs (57). Random measurement error can attenuate paths, whereas correlated error can inflate them; because an indirect association is a product of component paths, mediator measurement can materially change mediation conclusions (58). The apparent indirect-only pattern could therefore differ in magnitude or significance with multi-item latent measures. Future work should develop and validate separate multi-item measures of prospect, threat-related concealment, protective refuge, safety, and activity intention.

Sixth, all variables were self-reported in the same questionnaire at the same time point. Shared response format, context, and participants' implicit theories may inflate correlations, and the use of several single-item measures may exacerbate this risk. Harman's diagnostic is reported as a limited check, but it cannot exclude common method variance. Future studies should temporally separate predictor, mediator, and outcome measurement; combine self-report with objective scene features and observed behavior; and use independent or repeated assessments.

Seventh, the design was hybrid rather than a fully causal mediation experiment. Image assignment was randomized, but participants' appraisals, perceived safety, the PRS-11 score, and physical activity intention were measured cross-sectionally and were not experimentally manipulated. Temporal precedence cannot be established, unmeasured confounding cannot be ruled out, and alternative orderings—for example, restorative appraisal influencing safety or activity intention influencing global scene evaluation—may also fit the covariance structure (59). We therefore use associational language and recommend longitudinal or experimental tests of the mediator sequence.

Eighth, the study was designed to test mediation rather than moderation, so the questionnaire did not comprehensively measure candidate moderators. The sex and hukou sensitivity analyses do not address age, education, nature connectedness, prior outdoor experience, habitual physical activity, or image category. Likewise, pooling deliberately diverse scenes supported variation in the focal appraisals but does not establish equal pathways across all seven landscape categories. Balanced subgroup recruitment and preregistered moderation or category-specific studies are needed to determine for whom and in which natural settings the pathways apply.

## 5 Conclusions

Perceived prospect and protective refuge were positively associated with physical activity intention, whereas perceived threat-related refuge was negatively associated. The direct paths to physical activity intention became non-significant after perceived safety and perceived environmental restorativeness were included, yielding an indirect-only statistical pattern. The findings highlight that refuge is not uniformly positive or negative: it may be appraised as usable protection or as concealment for danger. For design practice, visibility, route legibility, threat reduction, and access to appropriate shelter may jointly support safer and more restorative activity settings. These cross-sectional appraisal data do not establish causal mediation, and the pathways require replication with validated multi-item measures, real environments, diverse populations, and observed physical activity.

## Data Availability

The raw data supporting the conclusions of this article will be made available by the authors, without undue reservation.
