# Supplementary material for: Associations between perceived natural environmental features and physical activity intention: the mediating roles of perceived safety and perceived environmental restorativeness
Source: Front Public Health. 2026 Sep 10;14:1892515. doi: 10.3389/fpubh.2026.1892515 (PMC13601344; doi:10.3389/fpubh.2026.1892515)
Supplement: Supplementary file 1 [file Data_Sheet_1.docx]

**Supplemental Materials**

**Figure S1.** Simulated natural landscapes for testing

Table S1. Exploratory factor analysis of the PRS-11 items (N = 1,439)

| **Item** | **Factor loading** | **Communality** |
| --- | --- | --- |
| PRS1 | 0.817 | 0.668 |
| PRS2 | 0.812 | 0.660 |
| PRS3 | 0.203 | 0.041 |
| PRS4 | 0.802 | 0.644 |
| PRS5 | 0.869 | 0.755 |
| PRS6 | 0.848 | 0.718 |
| PRS7 | 0.741 | 0.550 |
| PRS8 | 0.668 | 0.447 |
| PRS9 | 0.788 | 0.621 |
| PRS10 | 0.648 | 0.419 |
| PRS11 | 0.513 | 0.263 |

*Note. Principal axis factoring was applied to the Pearson correlation matrix. Parallel analysis (1,000 random datasets; 95th-percentile criterion) supported one factor; therefore, no rotation was applied. PRS-11 = 11-item Perceived Restorativeness Scale.*
